# Effect of HfC Addition on Microstructure and Wear Resistance of CoCrFeNiTi Coatings Fabricated by Laser Cladding

**DOI:** 10.3390/ma19050841

**Published:** 2026-02-24

**Authors:** Junbiao Zheng, Fangyan Luo, Xinnuo Li, Xuemeng Zhan, Chao Gao, Jiang Huang

**Affiliations:** 1 School of Electronics and Information Engineering, Guangdong Ocean University, Zhanjiang 524088, China; 2 Guangdong Provincial Key Laboratory of Intelligent Equipment for South China Sea Marine Ranching, Guangdong Ocean University, Zhanjiang 524088, China

**Keywords:** high entropy alloy, HfC, in situ TiC, wear resistance performance

## Abstract

HfC possesses high hardness, high melting point, and excellent thermal stability, and is regarded as an important wear-resistant reinforcing phase material. In this study, the laser cladding technique was employed to fabricate CoCrFeNiTi and CoCrFeNiTi/HfC composite coatings on the surface of Q235 substrate. The influence of HfC addition on the phase structure evolution, microstructure, and wear resistance of the coatings was systematically investigated. The results showed that the addition of HfC did not alter the phase structure of the coating, which remained dominated by an FCC solid solution. However, they induced the formation of an in situ TiC strengthening phase and reduced the brittle Laves phase content, thereby optimizing the coating’s toughness. At the same time, the coating transformed from columnar to equiaxed crystals, with significantly finer grains and further improved structural uniformity. Compared with the CoCrFeNiTi coating, the CoCrFeNiTi/HfC composite coating exhibited a more stable friction coefficient, a significantly lower wear rate, and improved wear resistance by approximately 2.4 times. The performance improvement was mainly attributed to the load-bearing strengthening and crack-pinning effect of the in situ TiC, the inhibitory effect of the reduction in the Laves brittle phase on adhesive wear, and the synergistic effect of Hf, which forms a stable oxidation-protective film during friction.

## 1. Introduction

Q235 steel is extensively employed in engineering structures and industrial machinery owing to its exceptional toughness, weldability, and cost-effectiveness. However, its susceptibility to wear and corrosion significantly constrains its service lifespan and reliability, particularly in harsh operating environments [1]. To mitigate surface degradation and enhance component durability, advanced additive manufacturing strategies have attracted significant attention for surface functionalization [2,3,4]. Among these, laser cladding has emerged as a premier surface modification technique. Distinguished by low dilution [5], a dense microstructure [6], and robust metallurgical bonding [7], laser cladding is particularly effective for fabricating high-performance wear-resistant coatings.

Since Yeh et al. proposed the concept of high-entropy alloys (HEAs), these materials have demonstrated great potential in the fields of wear resistance, corrosion resistance, and high-temperature service due to their characteristics such as the high-entropy effect, lattice distortion effect, and slow diffusion effect [8]. Among them, the CoCrFeNi alloy has been widely studied due to its excellent plasticity and corrosion resistance, and by adding Ti elements to construct the CoCrFeNiTi system, its strengthening ability and structural stability can be further enhanced [9,10]. However, in the rapid solidification and non-equilibrium environment of laser cladding, Ti promotes the precipitation of Laves-type brittle intermetallic compounds, leading to cracks and reducing the wear reliability of the coating [11].

To enhance the load-bearing capacity and wear resistance of the HEA coating, scholars generally adopt the method of adding ceramic reinforcing phases (such as VC, NbC, WC, etc.) to improve its comprehensive performance [12,13,14]. Gao et al. found [15] that the in situ reaction to generate TiC not only improves the tissue density, but also, by virtue of its high hardness, high stability, and excellent interface bonding properties, effectively withstands external loads, inhibits scraping wear, and refines grains, significantly enhancing the wear resistance of the coating. Zhou et al. confirmed [16] that the size and dispersion of TiC particles are crucial in determining the wear behavior. Smaller TiC particles enhance stability and wear resistance, while larger TiC particles and the brittleness of the coating reduce wear resistance. At the same time, by regulating the formation process of carbides, the content of brittle phases such as Laves can be suppressed to a certain extent, thereby improving the structural stability and friction-wear behavior of the coating.

In recent years, Hf, a refractory metal, has gradually attracted attention in the HEA system [17,18]. Hf has a large atomic radius and a strong carbon affinity, which can influence microstructure during cladding. To a certain extent, this improves the phase structure stability and service reliability of the alloy. Meanwhile, HfC, as a typical ultra-high-temperature ceramic material, has an extremely high melting point, excellent hardness, and thermal stability. Its addition is believed to play an important role in bearing external loads, inhibiting plastic deformation and wear evolution [19,20,21,22]. However, the systematic research on the microstructure evolution mechanism, in situ reaction behavior, and wear mechanism regulation effect of HfC reinforcing CoCrFeNiTi coatings is still relatively limited. Further exploration is urgently needed.

Based on these considerations, this study fabricated CoCrFeNiTi and CoCrFeNiTi/HfC composite coatings via laser cladding. It systematically elucidated the mechanisms underlying HfC addition’s influence on phase evolution and its enhancement of wear resistance. The results demonstrate that HfC incorporation not only regulates phase constitution and microstructural stability by promoting in situ TiC formation and suppressing brittle Laves phases, but also contributes to the formation of a stable oxide protective layer during sliding, thereby synergistically enhancing the tribological performance of the coatings. Furthermore, this work provides a quantitative assessment of the wear-resistance improvement induced by HfC, clarifying its reinforcing efficiency in laser-cladded CoCrFeNiTi coatings. These findings collectively establish the novelty of the present study from the perspectives of phase-regulation mechanisms, tribological response, and quantitative performance enhancement, offering theoretical insights and experimental support for the compositional design and engineering application of high-performance high-entropy alloy composite wear-resistant coatings.

## 2. Experimental Materials and Methods

### 2.1. Preparation of Experimental Materials

**Figure 1:**
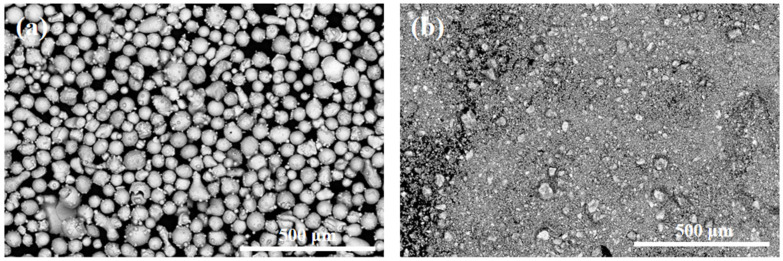
SEM images of the powders: (**a**) CoCrFeNiTi; (**b**) HfC.

Q235 low-carbon steel plates with dimensions of 100 mm (length) × 50 mm (width) × 3 mm (height) were used as the substrate material. Prior to laser cladding, the substrate surfaces were sequentially ground using 100-, 500-, 800-, and 1000-mesh sandpapers to remove surface oxides and contaminants. The polished substrates were then ultrasonically cleaned in ethanol and dried in a drying oven. The morphologies of the CoCrFeNiTi alloy powder and the HfC ceramic powder are shown in Figure 1. The CoCrFeNiTi alloy powder was commercially purchased from Sichuan Quanyue Metal Material Co., Ltd. (Ziyang, China). Meanwhile, the HfC ceramic powder was supplied by Hebei Yuanying New Materials Co., Ltd. (Xingtai, China). The nominal chemical compositions of the raw materials (mass fraction) are summarized in Table 1.

### 2.2. Mixing of CoCrFeNiTi/HfC Powders and Preparation of Coatings

The materials used for laser cladding consisted of a powder mixture containing 85 wt.% CoCrFeNiTi alloy powder and 15 wt.% HfC ceramic powder. The powders were first weighed according to the designed mass ratio and subsequently mixed in a planetary ball mill (Shandong Laiende Intelligent Technology Co., Ltd., Jinan, China). The ball milling process was conducted at a ball-to-powder ratio (BPR) of 10:1 and a rotational speed of 300 rpm for 2 h. During milling, a small amount of anhydrous ethanol was introduced as a process control agent (PCA) to improve powder dispersion and suppress excessive cold welding.

After ball milling, the mixed powders were dried in a forced-air drying oven (Shaoxing Shangyu Tianyu Plastic Instrument Factory, Shaoxing, China) at 80 °C for 24 h to completely remove residual ethanol. The dried powder mixture was then uniformly preplaced onto the substrate surface using a powder preplacement method, with the thickness of the preplaced powder layer precisely controlled at (2.0 ± 0.1) mm.

**Figure 2:**
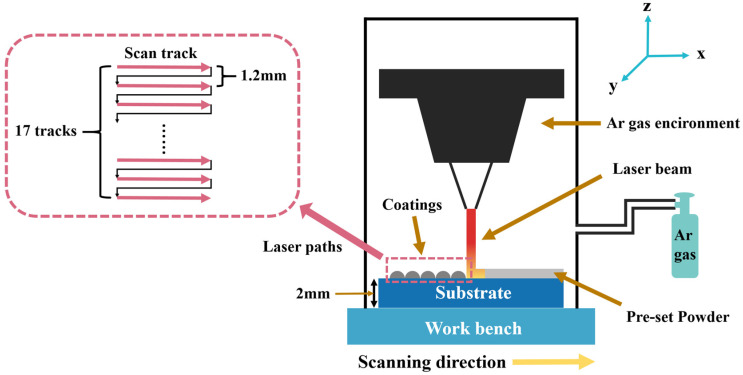
Schematic Diagram of Laser Cladding Process.

All coatings were fabricated using a single-pass laser cladding strategy, in which the coating layer was formed by successive overlapping tracks under identical processing parameters, without repeated remelting of the same region. Prior to laser cladding, the mixed powders were preplaced onto the substrate surface in a dry state using a custom-designed stainless-steel mold, with no binder or paste used during preplacement. The thickness of the preplaced powder layer was precisely controlled by the mold geometry and fixed at 2 mm, while the effective melting length during laser cladding was approximately 45 mm. Laser cladding was performed using a continuous-wave fiber laser system (XL-F2000W, Maxphotonics Co., Ltd., Shenzhen, China). Based on a series of preliminary experiments, the processing parameters were optimized as follows: 1800 W of laser power, 500 mm/min scanning speed, 50% overlap ratio, 5 mm defocusing distance, 17 cladding tracks, and a laser wavelength of 1080 nm. Throughout the cladding process, argon gas was continuously supplied as a shielding atmosphere at a flow rate of 5 L/min to effectively protect the molten pool and suppress oxidation. A schematic illustration of the laser cladding setup is provided in Figure 2.

## 3. Characterization

The phase composition of the coatings was characterized by high-resolution X-ray diffraction (XRD, XRD-6100, Shimadzu, Kyoto, Japan) using Cu–Kα radiation. The measurements were conducted under the following conditions: a scanning range of 20–90° (2θ), an operating voltage of 40 kV, and a continuous scanning mode with a scanning speed of 2°/min. The microstructure and elemental distribution of the coatings were examined by field-emission scanning electron microscopy (FE-SEM, ZEISS Sigma, Zeiss, Baden-Württemberg, Germany) coupled with energy-dispersive spectroscopy (EDS, Oxford 30Xplore, Oxford, UK).

The wear performance of the coatings was evaluated using a pin-on-disk tribometer equipped with a displacement sensor (SFT-2M, Lanzhou Zhongkehua Technology Development Co., Ltd., Lanzhou, China). A Si_3_N_4_ ball with a diameter of 4 mm was employed as the counterbody. The test parameters were set as follows: an applied load of 15 N, a rotation radius of 2 mm, a rotational speed of 200 rpm, and a testing duration of 30 min. After the wear tests, the wear tracks were scanned using the integrated three-dimensional profilometry system, and the wear volume (V) was directly measured. The wear rate (L) of the coatings was calculated based on Archard’s law [23], as expressed in Equation (1):

(1) $$L\;=\;V\;\times \;{\left(N\;\times \;d\right)}^{-1\;}$$

where V (mm^3^) is the wear volume directly measured from the experiment, N (N) is the applied normal load, and d (m) represents the total sliding distance. The total sliding distance (d) was calculated using the following Equation (2):

*d* = 2*πR* × *n* (2)

where R (m) is the sliding radius, and n denotes the total number of disk revolutions during the wear test. To ensure the reliability and reproducibility of the experimental results, each coating was subjected to at least three independent wear tests under identical conditions. The wear rate values reported in this study represent the average of repeated measurements.

## 4. Results and Discussion

### 4.1. Composition of Components

**Figure 3:**
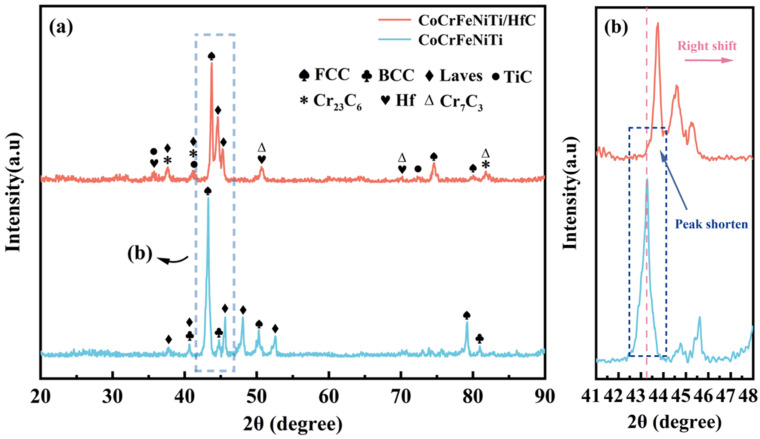
(**a**) XRD patterns of the CoCrFeNiTi and CoCrFeNiTi/HfC coatings; (**b**) enlarged image of 2θ within the range of 41° to 48°.

Figure 3a presents the XRD patterns of the CoCrFeNiTi alloy coating and the CoCrFeNiTi/HfC composite coating. The CoCrFeNiTi coating predominantly consists of an FCC solid solution phase (PDF #52-0513, PDF #04-0850), accompanied by a minor fraction of BCC (PDF #65-4116, PDF #34-0396) and Laves (PDF #15-0336, PDF #05-0719, PDF #05-0713) phases. This phase constitution is consistent with results previously reported by our group [24]. After the introduction of HfC, the phase constitution of the coating undergoes noticeable evolution. In addition to the FCC matrix phase, the cladded coating exhibits additional precipitated phases of Hf (PDF #38-1478) and TiC (PDF #32-1383), along with metallic carbides such as Cr_23_C_6_ (PDF #35-0783) and Cr_7_C_3_ (PDF #36-1482). It should be emphasized that the FCC solid solution in the CoCrFeNiTi coating and that in the CoCrFeNiTi/HfC composite coating share the same crystal structure. Meanwhile, their dominant constituent elements and local chemical environments may differ owing to elemental redistribution during laser cladding.

During laser cladding, the temperature of the molten pool can transiently exceed 2000 K. Under such high-temperature conditions, HfC may undergo partial thermal dissociation (Reaction (3)), releasing C atoms that subsequently participate in interfacial reactions. The liberated C atoms can further interact with Cr and Ti present in the molten pool, giving rise to a series of possible carbide-forming reactions, as described by Reactions (4)–(7).

HfC→Hf + C(3)

3Cr + 2C→Cr_3_C_2_ (4)

7Cr + 3C→Cr_7_C_3_ (5)

23Cr + 6C→Cr_23_C_6_ (6)

Ti + C→TiC(7)

**Figure 4:**
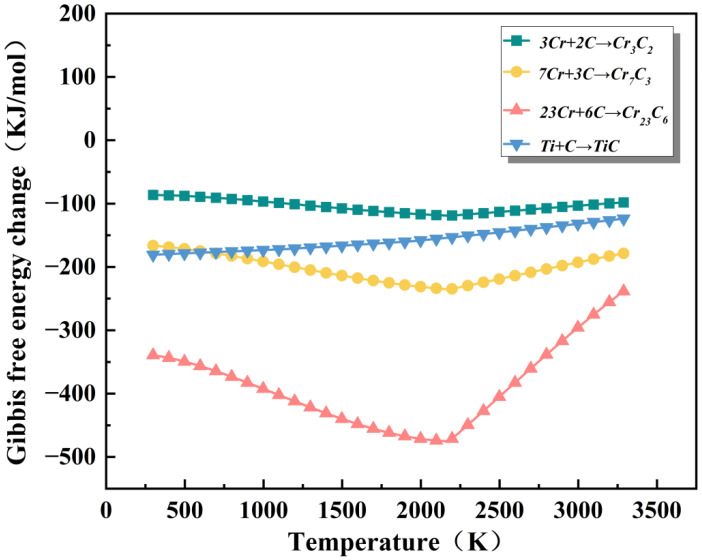
Graph showing the Gibbs free energy changes of different carbon materials at temperatures ranging from 298 to 3290 K.

To compare the thermodynamic driving forces of different carbide reactions, the relationship of Gibbs free energy with temperature was calculated. The results obtained using the HSC 6.0 software are shown in Figure 4. Within the temperature range of 298–3290 K, the Gibbs free energy ΔG of all reactions is negative, indicating that from a thermodynamic perspective, these carbide reactions all have the possibility of spontaneous occurrence [25]. However, the ΔG values of different reactions and their temperature trends vary significantly. The reaction for generating TiC maintains a relatively low Gibbs free energy throughout the temperature range, indicating that TiC has strong thermodynamic stability. Additionally, according to relevant references [24], TiC has an extremely high melting point (approximately 3290 K). It is more likely to form nuclei preferentially and remain stable under the rapid solidification conditions of laser cladding. Table 2 lists the ΔH_mix_ values between the elements in the coating. Existing studies [26,27] have found that a lower ΔH_mix_ indicates a higher solubility between the two elements. Hence, they are more likely to preferentially combine to form stable compounds. From our data, it can be seen that the ΔH_mix_ between Ti and C is lower than that between other elements and C, which further verifies our conjecture.

By enlarging the main diffraction peak region between 41° and 48° (as shown in Figure 3b), it can be clearly observed that, compared with the coating without HfC addition, the FCC main peak of the CoCrFeNiTi/HfC composite coating exhibits an evident shift toward higher diffraction angles, accompanied by peak broadening and a reduction in peak intensity. According to Bragg’s law [28], an increase in diffraction angle corresponds to a decrease in interplanar spacing, indicating a contraction of the lattice parameter of the FCC matrix. This behavior reflects an enhanced degree of lattice distortion within the FCC solid solution, which can be primarily attributed to the redistribution of solute elements induced by HfC addition, structural inhomogeneity formed under rapid non-equilibrium solidification conditions, and the associated residual stresses acting synergistically.

### 4.2. Microscopic Morphology

**Figure 5:**
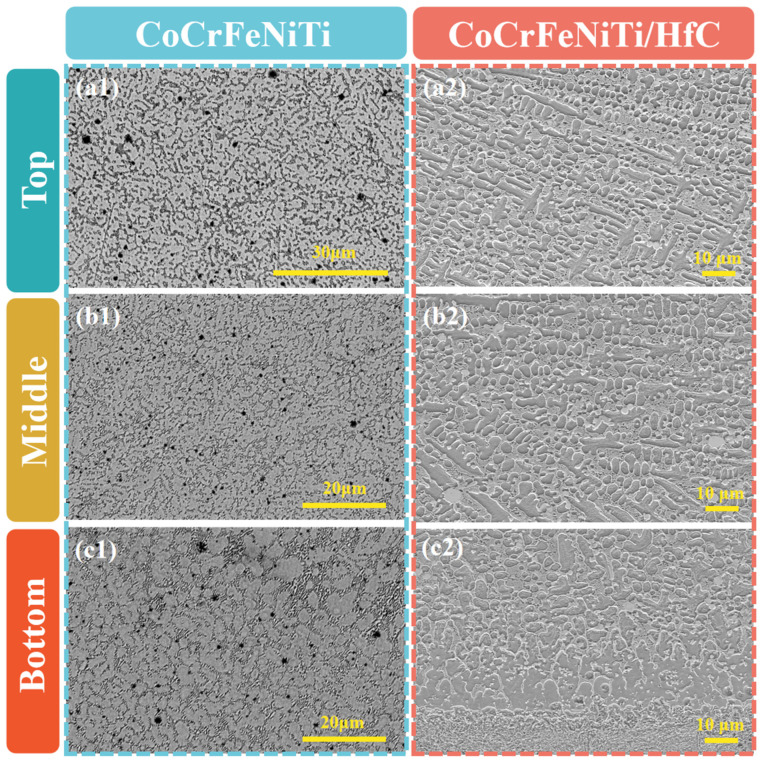
Cross-sectional SEM images of the CoCrFeNiTi and CoCrFeNiTi/HfC coatings at different heights. (**a1**–**c1**) CoCrFeNiTi coating at the top (**a1**), middle (**b1**), and bottom (**c1**) regions; (**a2**–**c2**) CoCrFeNiTi/HfC composite coating at the top (**a2**), middle (**b2**), and bottom (**c2**) regions.

Figure 5 presents representative cross-sectional SEM micrographs of the CoCrFeNiTi and CoCrFeNiTi/HfC coatings at different heights (Top, Middle, and Bottom). Both coatings exhibit dense and continuous microstructures throughout the coating thickness, with no visible cracks or pores, indicating good adaptability of the selected laser cladding parameters. The CoCrFeNiTi coating without HfC addition mainly consists of a uniformly distributed dark solid-solution matrix. By contrast, the introduction of HfC leads to the formation of numerous high-contrast white precipitates distributed along grain boundaries and within grains in the CoCrFeNiTi/HfC composite coating. These white precipitates are consistently observed at different heights, suggesting that they represent characteristic phases formed during microstructural evolution.

**Figure 6:**
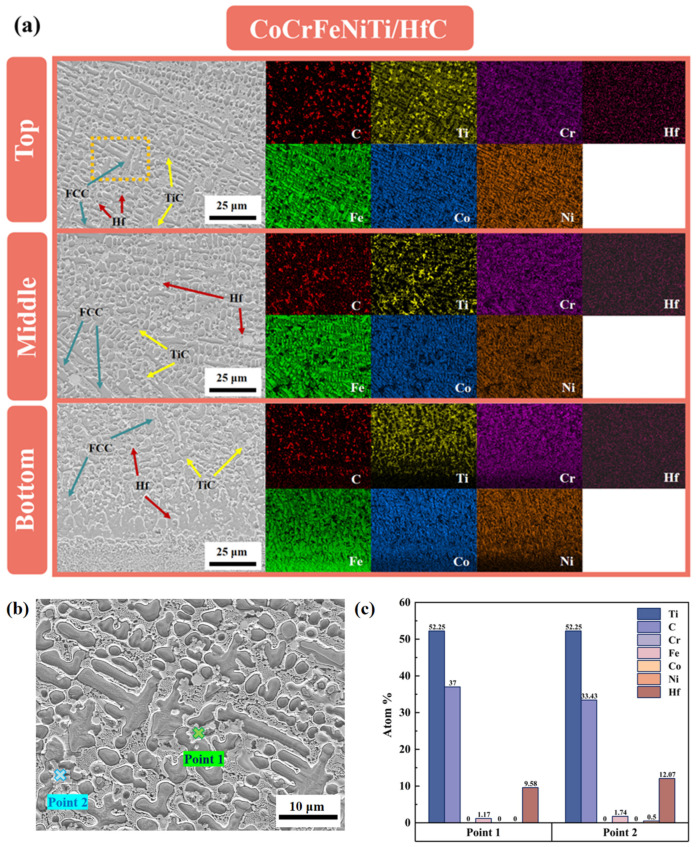
(**a**) Cross-sectional microstructure and corresponding EDS elemental maps of the CoCrFeNiTi/HfC composite coating at different heights (Top, Middle, and Bottom); (**b**) locally magnified microstructure of a representative region in the coating; (**c**) point EDS quantitative analysis results at the selected locations.

To further clarify the compositional characteristics of the white precipitates, Figure 6a presents cross-sectional microstructures of the CoCrFeNiTi/HfC composite coating at different heights, along with the corresponding EDS elemental maps. The white precipitated regions exhibit pronounced enrichment in Ti and C, accompanied by relative depletions of Co, Cr, Fe, and Ni, indicating that this phase corresponds to a Ti–C–enriched secondary phase. In addition, a locally magnified view of representative regions is provided in Figure 6b, where point analyses were conducted on the white precipitate (Point 1) and the adjacent matrix region (Point 2). As revealed by the quantitative EDS results in Figure 6c, Point 1 shows significantly higher atomic fractions of Ti and C than the surrounding matrix, whereas Point 2 remains dominated by Co, Cr, Fe, and Ni, which is consistent with the compositional characteristics of a high-entropy solid-solution matrix. When combined with XRD phase analysis, these results confirm that the dominant white precipitates in the CoCrFeNiTi/HfC composite coating are TiC.

**Figure 7:**
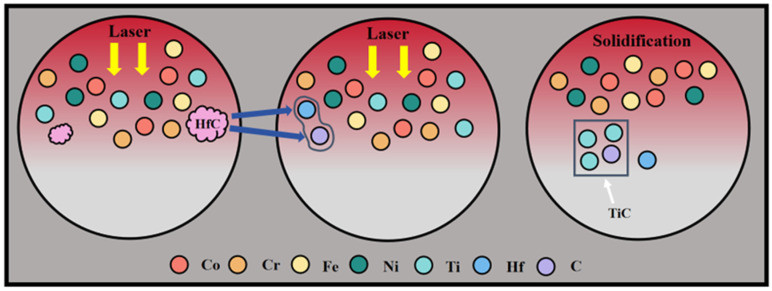
Phase evolution mechanism diagram of CoCrFeNiTi/HfC coating.

Previous studies have demonstrated that phase formation during laser cladding is strongly governed by elemental diffusion behavior [29]. In general, atomic size plays a critical role in diffusion kinetics, as a larger atomic radius corresponds to a higher diffusion energy barrier and reduced atomic mobility in the molten pool. As summarized in Table 3, the atomic radii of Hf and Ti are considerably larger than those of Co, Cr, Fe, and Ni, whereas C possesses the smallest atomic radius. During rapid solidification, smaller C atoms exhibit markedly higher diffusion rates, whereas the migration of larger atoms such as Hf is comparatively sluggish [30]. Although Ti has a larger atomic radius than the matrix elements, its strong chemical affinity for C favors the formation of thermodynamically stable carbides. Consequently, under the combined influence of kinetic diffusion and thermodynamic driving forces, highly mobile C atoms preferentially interact with Ti, leading to the in situ formation of fine TiC precipitates. This process is schematically illustrated in Figure 7, which depicts the preferential interaction between C and Ti atoms and the subsequent formation of TiC within the molten pool during laser cladding. It should be noted that, in the present system, CoCrFeNiTi alloy powder constitutes the primary component, resulting in a substantially higher concentration of Ti atoms than Hf atoms in the molten pool. Under the high-temperature molten-pool conditions dominated by rapid diffusion and stochastic atomic collisions, the statistical likelihood of Ti–C interactions is correspondingly increased, further promoting the preferential formation of TiC as the dominant carbide phase. The preferential formation of TiC also consumes Ti from the molten pool, thereby indirectly suppressing the formation of Ti-containing Laves phases, which is consistent with the reduced Laves-phase peak intensity observed in the XRD patterns of the CoCrFeNiTi/HfC coating.

**Figure 8:**
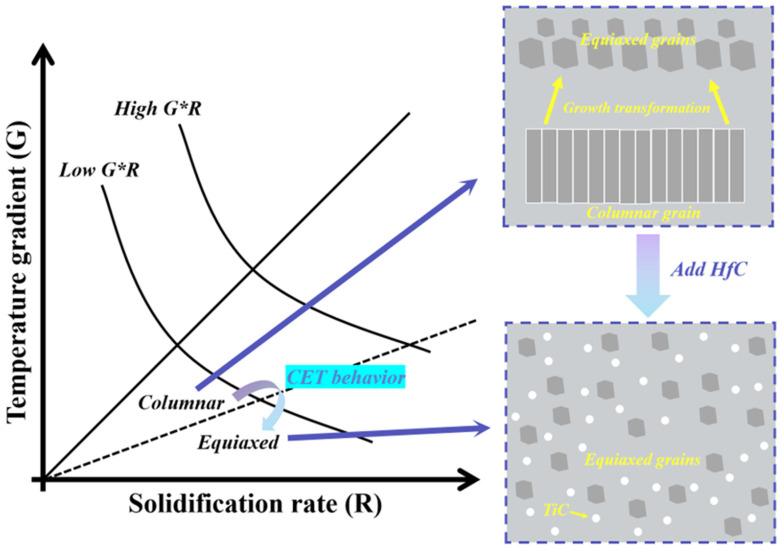
Schematic diagram of the evolution of grain structure.

Figure 8 schematically illustrates the evolution of grain structures during solidification. According to the classical G–R solidification theory [25,32], where G represents the temperature gradient, and R denotes the solidification rate, a high G combined with a low R favors heat-conduction-dominated solidification, promoting epitaxial growth along the heat-flow direction and resulting in columnar grain structures. As the value of G × R decreases, a columnar-to-equiaxed transition (CET) occurs, ultimately producing a refined equiaxed grain structure. In the present study, on the one hand, HfC particles serve as effective heterogeneous nucleation sites, significantly reducing the critical undercooling required for equiaxed grain formation and enhancing nucleation within the molten pool. On the other hand, the presence of HfC ceramic particles increases the melt viscosity, thereby weakening the stable epitaxial growth of columnar grains along the heat-flow direction. This combined effect effectively reduces the G/R ratio and promotes CET, resulting in pronounced grain refinement and equiaxed microstructures in the CoCrFeNiTi/HfC composite coating. These microstructural features are in excellent agreement with the SEM observations shown in Figure 5, further demonstrating that HfC ceramic reinforcements not only participate in phase formation but also play a critical role in regulating solidification microstructures.

### 4.3. Friction and Wear Resistance Performance

**Figure 9:**
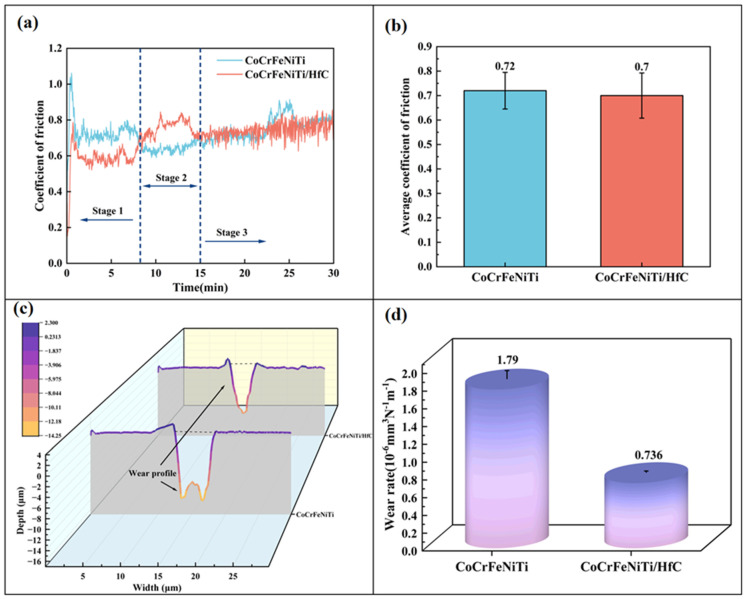
(**a**) Coefficient of friction; (**b**) Average coefficient of friction of the coating; (**c**) Wear profile of the coating; (**d**) Wear rate of the coating.

Figure 9a shows the coefficient of friction (COF) curves obtained when a Si_3_N_4_ ball with a diameter of 4 mm slid against the surfaces of the two coatings. The COF curves of both samples exhibit a degree of serrated fluctuation, consistent with observations reported by Li et al. [33]. It should be noted that the COF values presented in this study were calculated as time-averaged values based on continuously recorded friction data throughout the entire sliding test, thereby providing a representative description of the overall friction behavior of the coatings during the steady-state sliding stage. By comparing the two samples, it can be found that after 15 min, the CoCrFeNiTi/HfC shows a relatively stable COF, which reflects that adding 15% HfC can stabilize the COF curve of the coating to a certain extent. Figure 9b shows the average COF values of the two coatings. The average COF of the CoCrFeNiTi coating is 0.72, while that of the CoCrFeNiTi/HfC composite coating is 0.7. This result indicates that adding HfC effectively reduces the average COF and friction resistance. Figure 9c shows the wear profiles of the coating samples after 30 min. The test results show that the wear track of the CoCrFeNiTi coating has a wider and deeper width and depth, and the wear groove exhibits a clear irregular morphology, indicating severe material removal during the friction process. In contrast, the wear track of the CoCrFeNiTi/HfC composite coating is significantly shallower and its contour is gentler, indicating better wear resistance. Figure 9d shows the wear rates for each sample group, calculated according to the formula. The wear rate of the CoCrFeNiTi coating is 1.79 × 10^−6^ mm^3^ N^−1^ m^−1^, and the wear rate of the CoCrFeNiTi/HfC coating is 0.736 × 10^−6^ mm^3^ N^−1^ m^−1^. The wear resistance has been improved by approximately 2.4 times. This result further confirms the significant role of HfC particles in enhancing the wear resistance of the coating.

**Figure 10:**
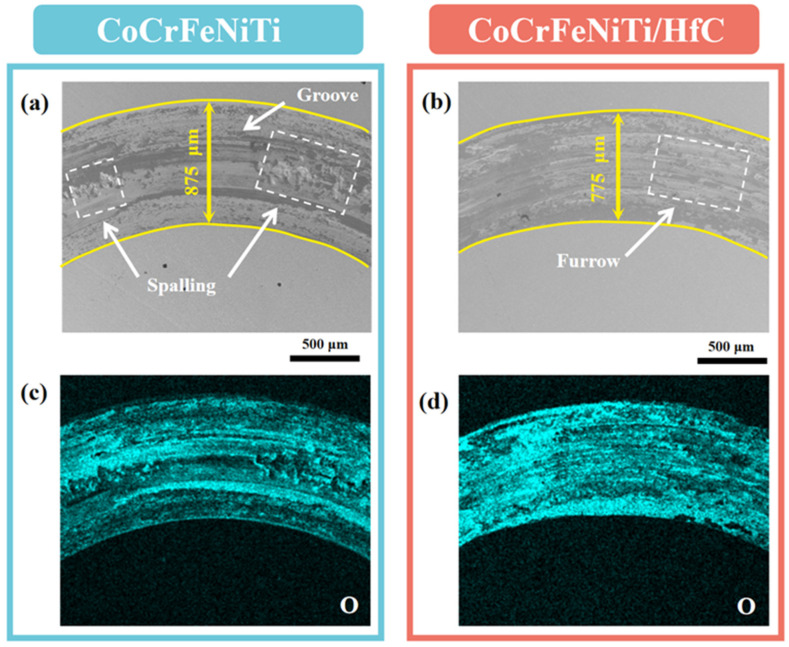
(**a**,**b**) SEM images of CoCrFeNiTi and CoCrFeNiTi/HfC coatings wear profiles; (**c**,**d**) O-element EDS surface scanning results of CoCrFeNiTi and CoCrFeNiTi/HfC coatings wear profiles.

**Figure 11:**
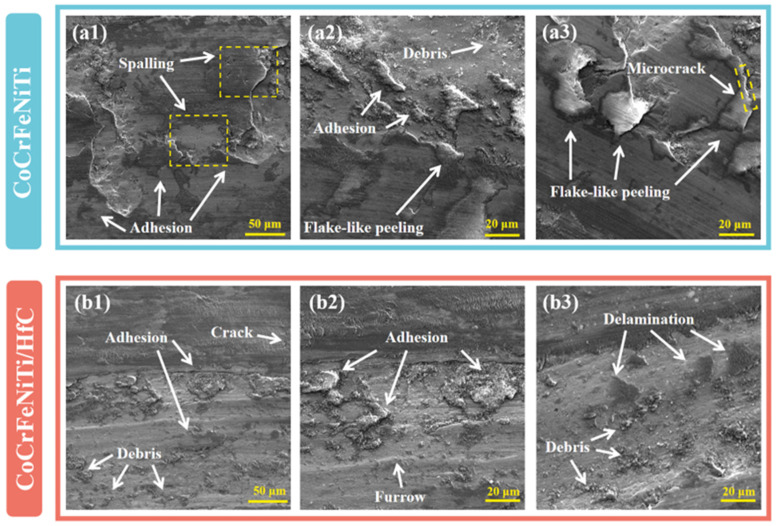
Local magnified SEM images of the worn surfaces of (**a1**–**a3**) CoCrFeNiTi coating and (**b1**–**b3**) CoCrFeNiTi/HfC composite coating.

To further elucidate the wear mechanisms, the worn cross-sections and local wear regions of the CoCrFeNiTi coating and the CoCrFeNiTi/HfC composite coating were systematically analyzed by combining wear profile morphology with EDS-based elemental distribution results, as illustrated in Figure 10 and Figure 11.

As shown in Figure 10a, the CoCrFeNiTi coating exhibits a relatively wide and deep wear track with an approximate width of 875 μm. Pronounced grooves and localized spallation features are clearly observed within the wear scar. Further examination of the locally magnified SEM images in Figure 11(a1–a3) reveals the presence of large spalled regions, lamellar delamination structures, and localized microcracks on the worn surface. These features can be mainly attributed to stress concentration at the contact interface between the Si_3_N_4_ counterbody and the coating during sliding, which induces cyclic plastic deformation of the surface layer and promotes crack initiation [34]. With continued sliding, the initiated cracks gradually propagate and coalesce, eventually leading to large-scale material detachment and fatigue-related damage [35]. In addition, a considerable amount of wear debris and adhesive traces can be observed on the worn surface (Figure 11(a2)), indicating pronounced material transfer during sliding. Taken together, these morphological characteristics suggest that the dominant wear mechanisms of the CoCrFeNiTi coating are adhesive wear accompanied by fatigue wear.

In contrast, the CoCrFeNiTi/HfC composite coating exhibits a markedly improved wear behavior. As shown in Figure 10b, the wear track width is significantly reduced to approximately 775 μm, and the overall wear scar appears smoother, with no evidence of large-scale spallation. The locally magnified SEM images in Figure 11(b1–b3) show that the worn surface of the composite coating is characterized by shallow ploughing grooves, limited wear debris, and localized adhesive traces, while only small-scale delamination and microcracks are observed in isolated regions. These observations indicate that the introduction of HfC effectively enhances the resistance of the coating to crack propagation and material detachment during sliding.

It is worth noting that the O-element EDS surface maps shown in Figure 10c,d reveal pronounced oxygen enrichment within the worn regions of both coatings, indicating that tribo-oxidation inevitably occurs during the sliding process. However, compared with the CoCrFeNiTi coating, the CoCrFeNiTi/HfC composite coating exhibits a more uniform and continuous oxygen distribution over the wear surface, suggesting a higher tendency to form a relatively stable tribo-oxide layer. This oxide layer can partially separate direct metal-to-metal contact and reduce adhesive interactions at the sliding interface, thereby synergistically mitigating the overall wear process.

**Figure 12:**
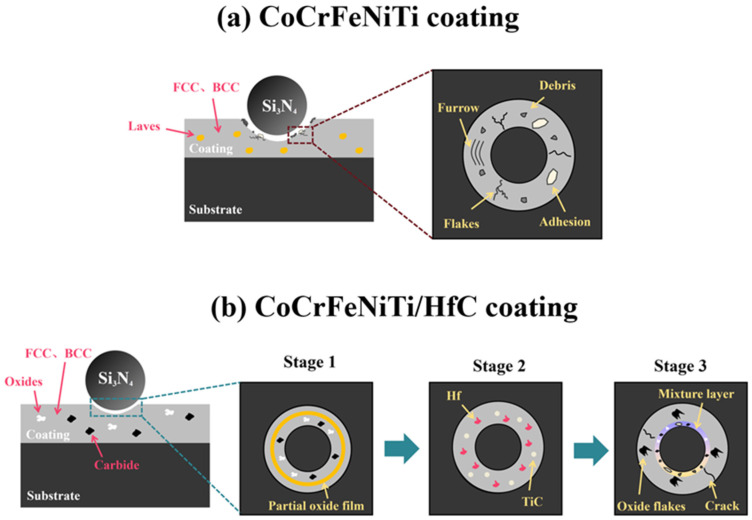
Wear mechanism diagram of (**a**) CoCrFeNiTi and (**b**) CoCrFeNiTi/HfC composite coatings.

Figure 12 schematically illustrates the wear mechanisms of the CoCrFeNiTi coating and the CoCrFeNiTi/HfC composite coating. During sliding, the CoCrFeNiTi coating exhibits evident spallation, localized cracking, and the generation of wear debris. In contrast, the wear process of the CoCrFeNiTi/HfC composite coating can be divided into three distinct stages. In the first stage, the contact between the Si_3_N_4_ ball and the coating surface is unstable due to the relatively small initial contact area, resulting in high local contact stresses. Under the combined effects of compressive loading and cutting action, the coating’s surface layer undergoes slight plastic deformation, generating a small amount of wear debris and leading to short-term fluctuations in the coefficient of friction (COF) [33]. As sliding proceeds, the wear surface is gradually smoothed and leveled, and the actual contact condition becomes more stable, causing a decrease in the COF. Meanwhile, frictional heating promotes oxidation reactions of surface-active elements, during which carbon atoms preferentially react with oxygen to form a tribo-oxide film [36,37]. This oxide film partially separates the counterbody from the coating substrate, thereby reducing direct contact and frictional resistance. Consequently, in the later part of the first stage, the COF further decreases, and the wear behavior is mainly governed by a combined adhesive–oxidative wear mechanism.

With further progression of wear, hard phases and the tribo-oxide layer in localized regions undergo fragmentation and partial detachment. The detached particles become trapped at the sliding interface and act as third-body abrasives, thereby introducing an abrasive wear component and causing a temporary increase in frictional resistance. Subsequently, under continuous shear and compressive stresses, part of the wear debris is crushed and redistributed or compacted onto the worn surface, forming a relatively stable tribological interface. As a result, the coefficient of friction decreases again. Therefore, the second stage is characterized by a COF evolution trend of initial increase followed by a decrease. Overall, the wear mechanism in this stage is dominated by adhesive wear, accompanied by a certain contribution from abrasive wear.

After entering the third stage, abrasive particles, flaking fragments, and oxides generated during the initial wear process are compressed and adhered to the worn surface under repeated extrusion and shearing actions, forming a relatively stable mixture layer that provides certain bearing and buffering functions for the friction interface. At the same time, the cracking and shedding of the surface oxide film cause a sudden change in the contact state between the ball and the coating, resulting in fluctuations in the friction resistance [38]; the shed oxides are quickly collected by the ball and the compressed worn surface and participate in the formation of the new phase layer, thus causing the COF to show small fluctuations at a stable level.

Based on the analysis of the wear morphology and friction coefficient evolution patterns mentioned above, it can be further concluded that the improvement in the wear resistance of the CoCrFeNiTi/HfC composite coating is not the result of a single factor. Firstly, during laser cladding, some Ti atoms react in situ with C atoms to form TiC particles, which exhibit high hardness and thermal stability. These TiC particles can effectively share the external load and enhance the coating’s resistance to the abrasive action of abrasive particles [39]. At the same time, the TiC particles form a strong metallurgical bond with the metal substrate, providing significant adhesion and refining the microstructure of the CoCrFeNiTi/HfC composite coating, effectively inhibiting the rapid expansion and penetration of cracks, thereby reducing fatigue spalling [40]. Zhou et al. reported [16] that TiC has good wetting properties and an excellent interface bonding force between the particles and the substrate. Hence, the CoCrFeNiTi/HfC composite coating has a better friction coefficient and wear rate. In addition, some fine TiC particles participate in the construction of new phase structures during friction, making the interface friction state tend to be stable.

Moreover, the in situ formation of TiC leads to a pronounced reduction in the content of the brittle Laves phase compared with the CoCrFeNiTi coating. As evidenced by the comparison between Figure 11(a1,b1), the spalled fragments observed on the CoCrFeNiTi coating are significantly larger. Although the Laves phase has relatively high hardness, it exhibits poor plasticity and high brittleness, making it highly susceptible to crack initiation and brittle fracture under cyclic loading. Consequently, the reduced fraction of the Laves phase effectively alleviates severe adhesive wear and spallation induced by brittle phase fracture. As a result, the wear behavior gradually transitions toward a more stable regime dominated by adhesive wear accompanied by mild abrasive wear.

Finally, Zhou et al. discovered that the incorporation of Hf could enhance the high-temperature wear resistance of HEA by altering the diffusion pathways of elements. At the same time, it promoted the inward diffusion of O, thereby facilitating the formation of a thinner protective oxide layer [18]. Therefore, during friction, as the interface temperature rises, the Hf element participates in a frictional chemical reaction, generating Hf-containing oxides with greater stability. Such frictional chemical films can form a relatively dense protective layer at the wear interface, providing isolation and buffering effects, reducing the direct contact between the ball and the coating substrate, and thereby inhibiting adhesive wear.

## 5. Conclusions

In this study, the laser cladding technology was employed to successfully fabricate CoCrFeNiTi and CoCrFeNiTi/HfC composite coatings on the surface of Q235 base material. The strengthening mechanism of HfC was systematically analyzed based on the microstructure evolution, phase structure characteristics, and friction and wear behaviors. The main conclusions are as follows:

(1) Both coatings exhibited excellent forming quality, with dense structures and no obvious defects. The CoCrFeNiTi coating is mainly composed of an FCC solid solution and a small amount of Laves phase. The addition of HfC did not change the overall structural features of the coating, which remained mainly FCC, but induced the formation of an in situ TiC strengthening phase, while weakening the peak strength of the Laves brittle phase.
(2) The microstructure analysis shows that the grains of the CoCrFeNiTi/HfC composite coating have transformed from columnar to equiaxed, with significant grain refinement and a remarkable improvement in microstructural uniformity. The in situ TiC particles are dispersed at grain boundaries and within grains, forming a strong metallurgical bond with the matrix. They perform significant load-bearing strengthening, crack pinning, and deflection, effectively inhibiting crack initiation and propagation.
(3) Friction and wear resistance tests show that adding HfC significantly improves the material’s wear resistance. Compared with the CoCrFeNiTi coating, the CoCrFeNiTi/HfC composite coating exhibits a more stable friction coefficient, a significantly reduced wear rate, and improved wear resistance by approximately 2.4 times. The performance improvement is mainly attributed to: the in situ-generated fine TiC particles with high hardness and crack-inhibition functions; the reduction in the Laves brittle phase leading to weaker adhesive wear and brittle shedding; and the synergistic effect of Hf, which forms a stable oxide protective film during friction.

In summary, this work provides a systematic understanding of the phase evolution and strengthening mechanisms of CoCrFeNiTi/HfC composite coatings fabricated by laser cladding, mainly from a thermodynamic and experimental perspective. It is acknowledged that laser cladding is a highly dynamic and non-equilibrium process, in which phase formation is governed by the combined effects of thermodynamic driving forces and kinetic factors. While the present study focuses on revealing the dominant thermodynamic tendencies and their correlation with the observed microstructures, a more quantitative consideration of kinetic effects and process variability remains an important topic for future research. Further studies integrating thermodynamic and kinetic perspectives are expected to deepen the understanding of phase evolution under practical laser cladding conditions.

## Figures and Tables

**Table 1:** Chemical compositions of materials (mass fraction%).

Material	Chemical Composition (Mass Fraction)										
	C	Si	Mn	Fe	S	P	Cr	Co	Ni	Ti	Hf
Q235	0.15	0.15	0.25	Bal.	0.11	0.12	-	-	-	-	-
CoCrFeNiTi	-	-	-	20.64	-	-	19.06	21.3	21.45	17.55	-
HfC	6.3	-	-	0.15	-	-	-	-	-	-	Bal.

**Table 2:** Mixed enthalpy ΔH_mix_ (kJ/mol) between elements [24,28].

ΔH_mix_ (KJ/mol)	Co	Cr	Fe	Ni	Ti	C
Co	/	−4	−1	0	−28	−42
Cr		/	−1	−7	−7	−61
Fe			/	−2	−17	−50
Ni				/	−35	−39
Ti					/	−109

**Table 3:** Atomic radii of different elements [31].

Element	Co	Cr	Fe	Ni	Ti	Hf	C
Atomic radius (Å)	1.25	1.28	1.26	1.24	1.47	1.59	0.77

## Data Availability

The original contributions presented in this study are included in the article. Further inquiries can be directed to the corresponding authors.
